# A Preliminary Study on the Therapeutic Role of γδT Cells in Triple‐Negative Breast Cancer

**DOI:** 10.1002/kjm2.70029

**Published:** 2025-04-28

**Authors:** Shi Qiu, Qin‐Yu Han, Xian Zhao, Wen‐Jing Li, Xiang‐Qi Li

**Affiliations:** ^1^ Department of Oncology Affiliated Hospital of Jiangnan University Wuxi Jiangsu China; ^2^ Department of Breast Center The Second Affiliated Hospital of Shandong First Medical University Tai'an Shandong China

**Keywords:** adoptive immunity, breast cancer, gamma delta T (γδT) cell, immunotherapy, triple‐negative breast cancer

## Abstract

This study was aimed to elucidate the cytotoxic effects of γδT cells on triple‐negative breast cancer (TNBC) cells and assess their antitumor efficacy in a mouse xenograft model. Furthermore, the underlying mechanisms of γδT cell action on TNBC were explored. The study utilized three TNBC cell lines (MDA‐MB‐231, MDA‐MB‐468, and BT‐549) as target cells, with γδT cells serving as effector cells. Cytotoxicity was assessed in different effector‐to‐target ratios (E:T) at 5:1, 10:1, and 20:1 subsequent to coculture. To evaluate the antitumor effects of γδT cells in vivo, a xenograft mice model was established by inoculating MDA‐MB‐231 cells into the mammary fat pad of B‐NDG mice. The mice received tail vein injections of γδT cells at different doses. The effects on tumor growth, mouse body weight, and γδT cell accumulation in the spleen were then determined. γδΤ cells at E:T of 10:1 exhibited significant cytotoxicity against all three TNBC cell lines, indicating a statistically significant difference compared to the control group (*p* < 0.0001). The cytotoxic effect at this ratio was superior to that at 20:1 and 5:1 effector‐to‐target ratios, as evidenced by statistical significance (*p* < 0.05). Following 21 days of adoptive transfer via tail vein injection, γδΤ cells at both low and high doses significantly reduced tumor volume and mass compared to the PBS control group (*p* < 0.001). This reduction was accompanied by an increased accumulation of γδΤ cells in the spleen. In conclusion, γδΤ cells exert significant cytotoxic effects on TNBC cells and effectively inhibit the growth of breast cancer xenografts in mice while also promoting the accumulation of γδΤ cells in the mouse spleen.

## 1 Introduction

Triple‐negative breast cancer (TNBC) is a specific subtype of breast cancer characterized by the absence of three key receptors, including estrogen receptor, progesterone receptor, and human epidermal growth factor receptor 2 (HER‐2). It accounts for approximately 15%–20% of all breast cancer cases. TNBC is mostly recognized by its heterogeneity, rapid progression, invasiveness, and poor prognosis compared to other breast cancer types [1, 2, 3, 4].

TNBC as a frequent subtype of breast cancer is known for its extreme aggressiveness and poor outcomes due to its distinct immunophenotype. TNBC exhibits distinct biological features, including poor differentiation and genetic instability, when compared to other non‐TNBCs. Therefore, its unknown underlying pathogenesis necessitates more attempts to develop targeted treatment strategies. Currently, chemotherapy remains the sole effective pharmaceutical intervention. However, owing to its unique characteristics, chemotherapy often fails to effectively eliminate the tumor and may cause serious adverse side effects in patients.

TNBC cells exhibit significant heterogeneity and selective gene locus mutations across subtypes, potentially making them more antigenic and capable of effectively stimulating the immune system [5]. Theoretically, activating the immune response in the tumor microenvironment can lead to tumor cell destruction, suppression of tumor growth, and prevention of metastasis. Therefore, overcoming the body's tumor immune tolerance is the primary focus of current TNBC immunotherapy research [6]. The remarkable success rate of immune checkpoint inhibitors (ICIs), especially those targeting PD‐1/PD‐L1 and CTLA‐4, in treating diverse solid tumors offers new possibilities for TNBC immunotherapy, as monotherapy or combined with chemotherapy [7, 8]. The combination of the PD‐L1 inhibitor atezolizumab with albumin‐bound paclitaxel, compared to albumin‐bound paclitaxel alone, that was assessed as a first‐line treatment for metastatic or locally advanced TNBC resulted in improved survival outcomes [9]. Likewise, neoadjuvant therapy using pembrolizumab combined with chemotherapy enhanced the pCR rate and event‐free survival rate in early TNBC cases [10]. Nevertheless, PD‐L1 positive expression may be a good prognostic biomarker [11]. However, the response to PD‐1/PD‐L1 antibodies is still suboptimal, highlighting the need to discover novel immune checkpoint targets to enhance the efficacy of PD‐1/PD‐L1 blocking therapy.

As we know, there is no standardized treatment protocol specifically targeting TNBC, which complicates management and highlights the urgent need for new therapeutic strategies. Various therapeutic strategies comprise classic treatment approaches, including neoadjuvant and adjuvant chemotherapy, surgical options, and postsurgery radiation therapy [12, 13]. Currently, immunotherapy along with the mentioned traditional approaches has been introduced as an emerging treatment option for TNBC patients [14]. Immunotherapy, particularly through the use of ICIs, has made a significant transformation toward the treatment landscape for TNBC [15, 16]. This approach harnesses the power of the body's own immune defenses to recognize and combat cancer cells, which is particularly significant given the unique features of TNBC. It is aimed at stimulating or regulating the immune system to improve or reinforce immunity and the function of the tumor microenvironment, resulting in tumor immune suppression, and enhancing the cytotoxic effect on tumor cells [17, 18, 19].

The tumor microenvironment comprises heterogeneous and complex components and various cell types, including cancer, stromal, and immune cells, all of which play significant roles in tumor progression and response to therapy [20]. Communication between tumor cells and the immune microenvironment is proposed as a determining factor, particularly concerning its implications for successful immunotherapy [21] However, the presence of tumor antigens is a prerequisite for the activation of antigen‐specific T cells, which are crucial for effective immune responses against cancer [21, 22]. A large number of tumor‐infiltrating T lymphocytes have been identified in TNBC tissues; however, most of them are in an exhausted state, which may be closely related to the immunosuppressive microenvironment present in the majority of TNBC cases [18, 23].

Based on their T cell receptors (TCRs), T cells are primarily categorized into two groups: αβT and γδT cells with the approximate ratio of 95% and 5%, respectively. Typically, αβT cells display CD4+ and CD8+ markers and are predominantly classified as helper or cytotoxic effector subsets. In contrast, γδT cells generally are specialized in recognizing glycolipids and other small molecular compounds, including certain soluble peptide molecules. Interestingly, γδT cells are not constrained by major histocompatibility complex molecules and exhibit innate immune characteristics. These unique properties enable γδT cells to play vital roles in anti‐infection and antitumor responses [24]. Activated γδT cells are important candidates for cancer treatment since they can pose cytotoxic effects on tumor cells through various pathways both in vitro and in vivo. However, their efficacy in TNBC is still under investigation [25, 26]. In hypothesis, immunotherapy, mainly based on γδT cells, is expected to be a new treatment option for TNBC [27]. This study intends to explore the cytotoxic effect of γδT cells on TNBC cells and the growth of mouse xenograft tumors, aiming to preliminarily analyze the mechanism of action and provide experimental evidence for the treatment of TNBC.

## 2 Materials and Methods

### 2.1 Collection, Expansion, and Detection of γδΤ Cells

Peripheral blood samples from healthy volunteers were mixed with an equal volume of PBS and then diluted with Ficoll lymphocyte separation medium (Solarbio, product number P8610) at a 4:3 ratio. The tubes were centrifuged (18°C–20°C and 500 g for 30 min). After centrifugation, the white film layer was carefully transferred into a 50 mL sterile centrifuge tube, resuspended in PBS, and washed twice through centrifugation (300 g for 10 min).

Initial culture medium for γδΤ cell containing Zoledronic acid (Zol) (Servicebio, Wuhan, China), recombinant human interleukin‐2 (IL‐2) (Beijing Tongli Haiyuan Biotechnology Co), GT‐T551 H3 medium (Baoribio‐biotechnology, Beijing), and PBS was prepared as 0% FBS, IL‐2100 IU/mL, Zol 50 μmol/mL GT‐T551 H3. The pellet was resuspended in γδΤ cell initial culture medium. The cells were seeded in a 24‐well plate at a density of 3 × 10^6^ cells/well, and in a 6‐well plate at a density of 3 × 10^7^ cells/well, and were incubated in a cell culture incubator containing 5% CO_2_ at 37°C. After two weeks, the expansion of γδΤ cells was detected and subjected to purification using flow cytometry sorting. The sorting instrument was connected, tested, and used to sort the prestained cells. Flow cytometry was used to detect the purity of γδΤ cells postsorting.

### 2.2 Flow Cytometry Detection of γδT Cells

Approximately 1–3 × 10^6^ cells were collected and centrifuged (1500 rpm for 5 min). The supernatant was removed, and PBS (1 mL) was added to the pellet. The pellets were resuspended in 50 μL PBS and 5 μL each of TCR γ/δ PE (Biolegend, product number 331209) and CD3 PerCP (Biolegend, product number 300325) antibodies. The mixture was gently mixed and incubated for 20 min away from light. The cells were washed with PBS through centrifugation (500 rpm for 5 min) and the supernatant was discarded. Then the cells were resuspended in 500 μL PBS and tested on the flow cytometer (BD Company, USA) for purity.

### 2.3 Culture of Target Cells and Viability Assessment

TNBC cell lines, including MDA‐MB‐231, MDA‐MB‐468, and BT‐549 (Wuhan Procell Life Technology Co. China), were seeded into separate cell culture flasks according to the instructor recommended protocol. MDA‐MB‐231 and MDA‐MB‐468 were cultured in DMEM (Wuhan Procell Life Technology, China) with high‐glucose medium containing 10% FBS (Thermo Fisher, Carlsbad, CA). BT‐549 cell line was cultured in RPMI 1640 medium (American Hyclone, USA) containing 10% FBS, in a 37°C, 5% CO_2_ constant temperature cell culture incubator. Once the cells reached confluence, they were promptly passaged. TNBC cells MDA‐MB‐231, MDA‐MB‐468, and BT‐549 were collected at their growth phase, washed twice with PBS, centrifuged (1000 rpm for 5 min), counted, and then resuspended in GT‐T551 H3 medium containing 1% FBS.

The experimental groups were included (1) Group A: Cell lines that were incubated with an equal amount of PBS; (2) Group B: Cell lines that were incubated with γδΤ cells at a 5:1 effector‐to‐target ratio; (3) Group C: cell lines that were incubated with γδΤ cells at a 10:1 effector‐to‐target ratio; (4) Group D: Cell lines that were incubated with γδΤ cells at a 20:1 effector‐to‐target ratio. Cell viability was tested daily using the CCK‐8 assay kit (KEYGEN, product number KGA317) according to the manufacturer's instruction.

### 2.4 Study on TNBC Xenograft Mice Model

Following an in vitro experiment, a total of 15 female B‐NDG mice in their 8th week of age (Beijing Biocytogen Co. and Jiangsu Gene Biotechnology Co., license number: SCXK (Su) 2021–0005) were chosen for the in vivo study. MDA‐MB‐231 cells in their logarithmic growth phase were utilized. Approximately 1 × 10^7^ MDA‐MB‐231 cells were digested, suspended, and then injected into the fat pad under the third pair of nipples on the left side of the B‐NDG mice. Each mouse was individually identified by ID number and housed in an SPF environment, and daily monitoring was performed. The presence of a lump at the injection site at 10–14 days after injection indicated successful model construction. The 14th day was considered the first day of treatment.

Mice were randomly divided equally into three groups: PBS control group, low‐dose γδΤ cell group, and high‐dose γδΤ cell group, with 5 mice per group. All mice were housed under standard conditions and received tail vein injections of either PBS, 5 × 10^7^ sorted γδΤ cells per mouse (low dose), or 10 × 10^7^ sorted γδΤ cells per mouse (high dose) on days 1, 7, and 14 of the treatment.

During the treatment period, the mental state and hair condition of the mice, along with any adverse reactions such as diarrhea and allergies, were monitored. Body weight and tumor volume were recorded every 3 days. The length and diameter of the tumor were measured with a vernier caliper, and the volume was calculated using the formula *V* = (length×width^2^)/2. The mouse body weight change and tumor growth curves were plotted. On the 21st day of treatment, after the final body weight measurement, the mice were anesthetized, fixed on a wax board, and their tumor tissue was dissected for analysis. The therapeutic effect was assessed by comparing the tumor volumes across groups after 21 days of adoptive transfer.

### 2.5 Statistical Methods

Statistical analysis and data processing was performed using SPSS 22.0 statistical software, and the results were considered as mean ± standard deviation (mean ± SD). When the experimental data was normally distributed, for analyzing the homogeneity of variance, one‐way analysis of variance (ANOVA) was used to compare data differences between multiple groups, while *t*‐tests were employed to assess statistical differences between two groups. Statistical significance was set at *p* < 0.05. Graphs were plotted using GraphPad Prism 9.0 software.

## 3 Results

### 3.1 In Vitro Expansion of γδΤ Cells Using a Combination of Zoledronic Acid and IL‐2

**FIGURE 1:**
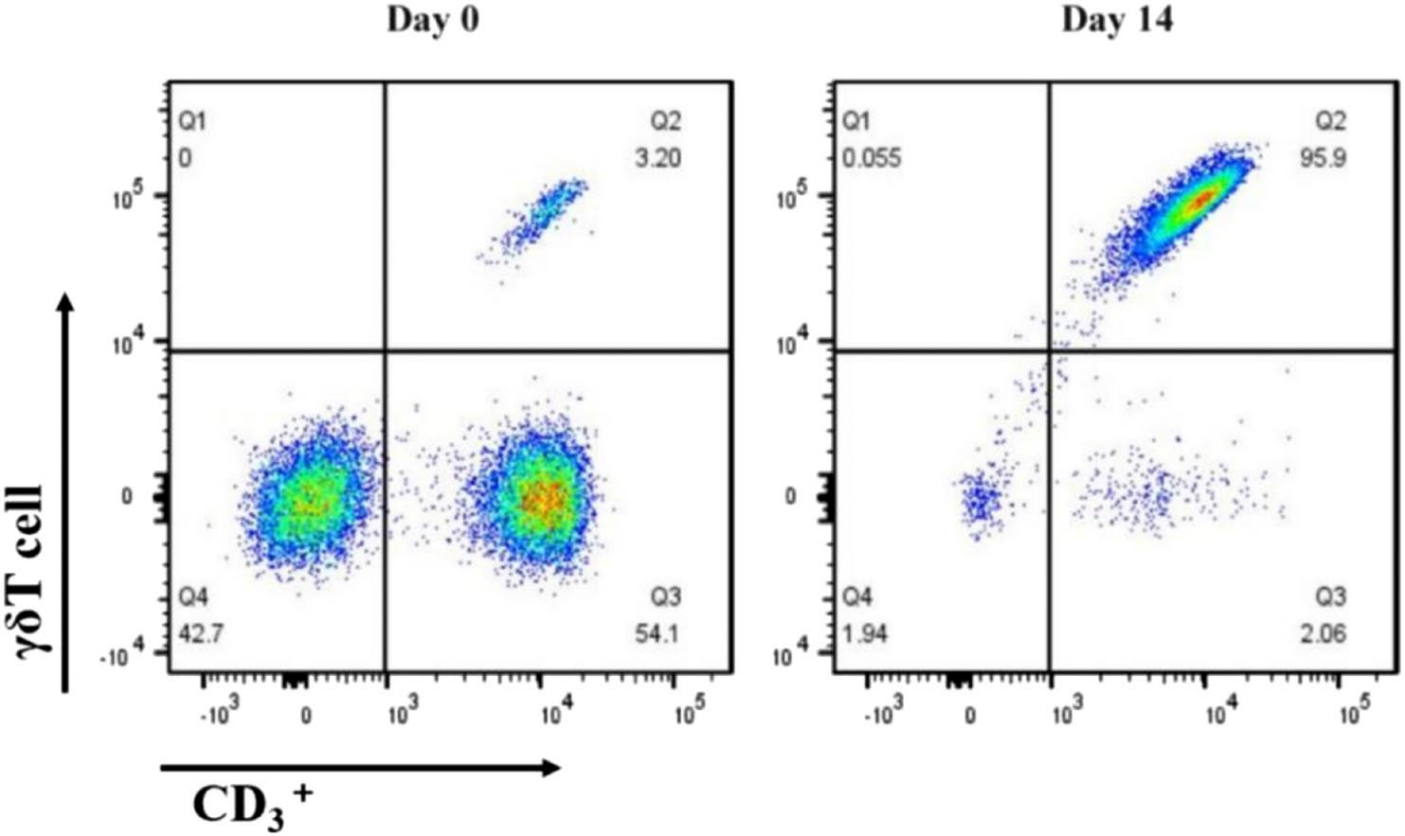
The in vitro results of the effect of zoledronic acid combined with IL‐2 on the expansion of γδΤ cells.

**FIGURE 2:**
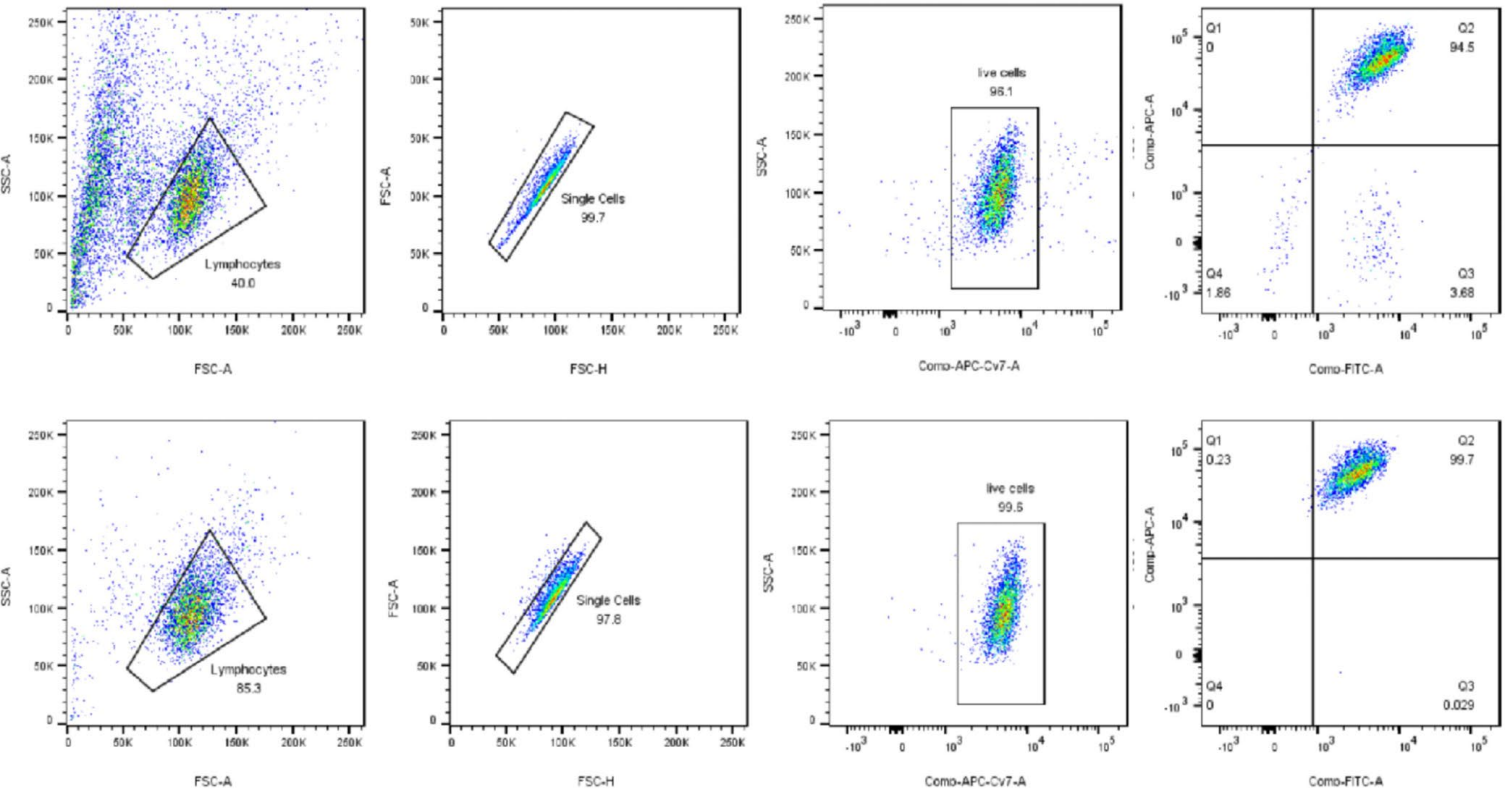
In vitro purification of γδT cells.

In vitro culture of freshly isolated peripheral blood mononuclear cells led to a significant proliferation of γδΤ cells over two weeks. By the end of this period, γδΤ cells constituted over 90% of viable cells, increasing from 1.83 × 10^4^ to 5.26 × 10^6^. This expansion corresponded to a substantial rise in the proportion of γδT cells from 3.67% to 92.97% of the viable cell population (Figure 1). Postsorting purity of γδΤ cells was reached up to 99% (Figure 2).

### 3.2 Cytotoxicity of γδΤ Cells Against TNBC

**FIGURE 3:**
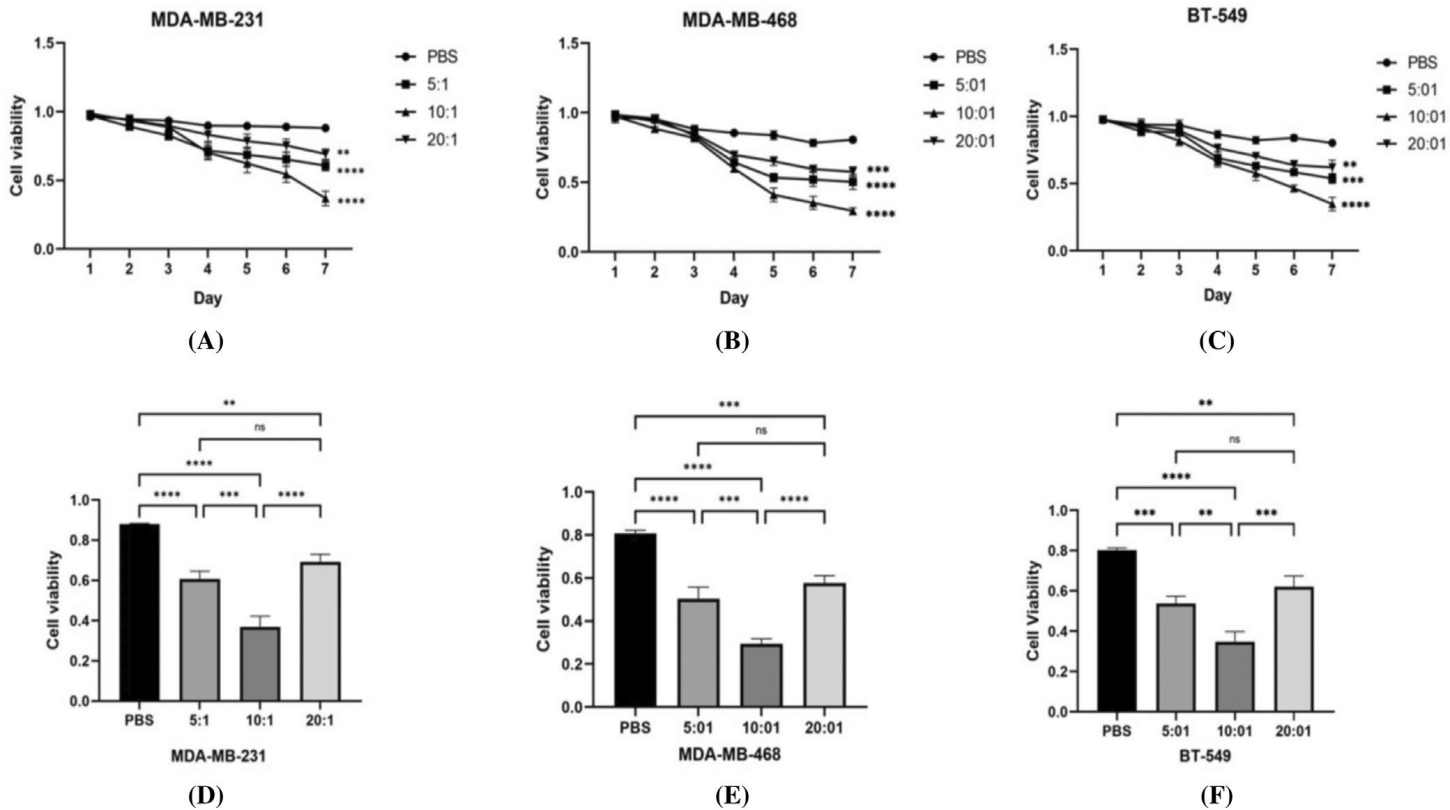
Cytotoxic activity of γδΤ cells against triple‐negative breast cancer cells in vitro. [(A–C) are the trend charts of the survival rate of tumor cells in each group within seven days detected by CCK‐8, and (D–F) are the seventh‐day survival rates of tumor cells in each group. Day survival rate, *p < 0.05, **p < 0.01, ***p < 0.001, ****p < 0.0001].

The cytotoxic effects of γδΤ cells on TNBC cells were evaluated at different effector‐to‐target (E:T) ratios in vitro. The results demonstrated that γδΤ cells at an E:T ratio of 10:1 effectively eliminated TNBC cells. This cytotoxicity was significantly greater than that observed in the PBS control group (*p* < 0.0001). Interestingly, γδΤ cells at an E:T ratio of 10:1 exhibited stronger cytotoxicity against MDA‐MB‐231 cells compared to the 5:1 and 20:1 ratios (*p* < 0.001 and *p* < 0.01 *respectively*). Similarly, the cytotoxicity of γδΤ cells at an E:T ratio of 10:1 on MDA‐MB‐468 cells was considerably higher than the PBS control group (*p* < 0.0001), whereas cytotoxicity at ratios of 5:1 and 20:1 was weaker and not significantly different (*p* > 0.05). Furthermore, γδΤ cells at an E:T ratio of 10:1 demonstrated significantly greater cytotoxicity against BT‐549 cells compared to ratios of 5:1 and 20:1 (*p* < 0.01 or *p* < 0.001), and their cytotoxicity effect on BT‐549 cells was also substantially higher than that of the PBS control group (*p* < 0.0001). While the cytotoxicity of γδΤ cells at 5:1 and 20:1 ratios against BT‐549 cells was significantly greater than that of the PBS control group (*p* < 0.001, *p* < 0.01), it was less potent than that observed at an E:T ratio of 10:1, with no significant difference between the 5:1 and 20:1 ratios (*p* > 0.05) (Figure 3).

### 3.3 Effect of γδΤ Cells on TNBC Xenograft Mice Model

**FIGURE 4:**
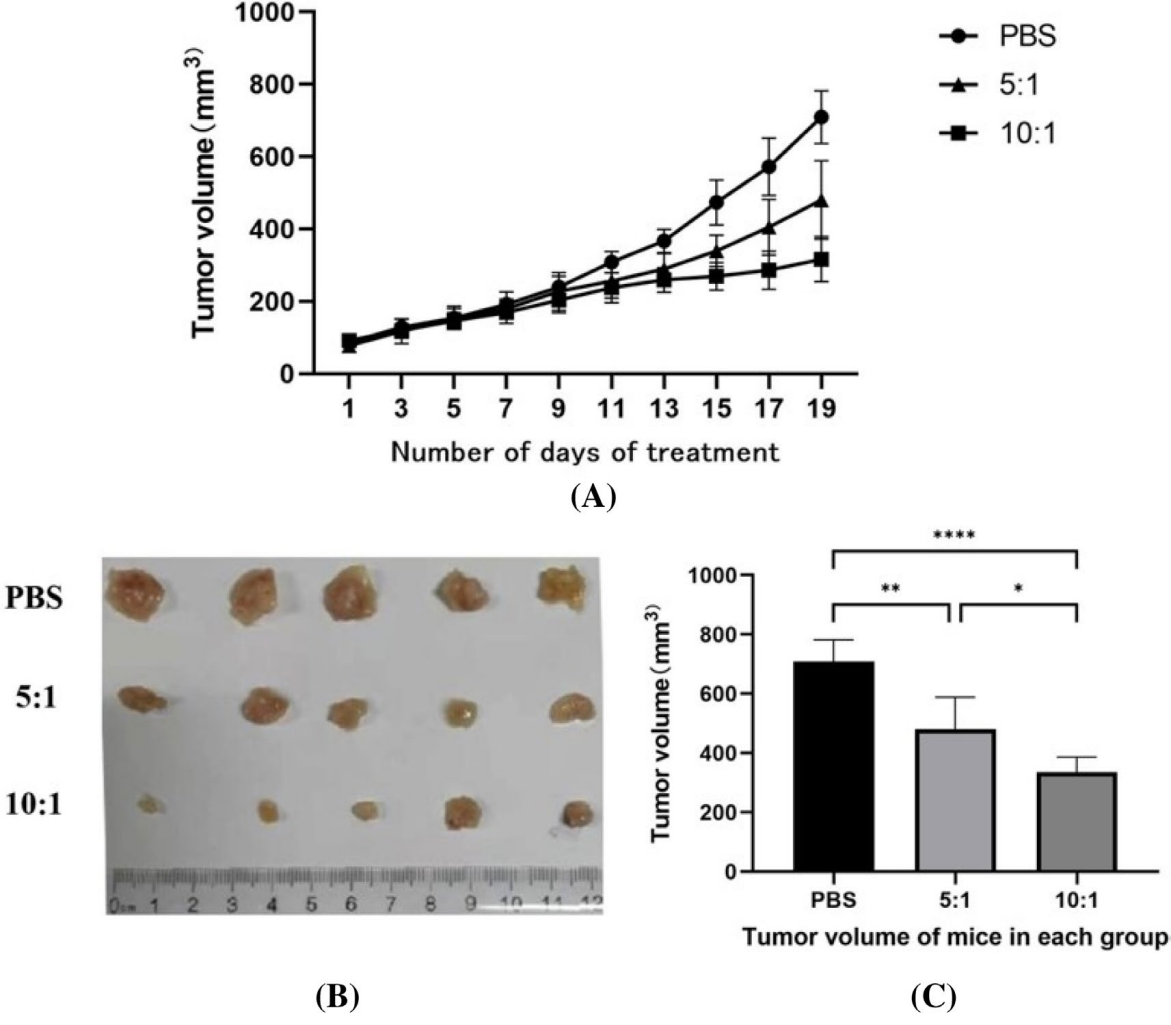
Inhibitory effect of γδΤ cells on transplanted tumors in mice. [(A) is the growth trend of tumor volume in each group after treatment, (B) shows the dissected transplanted tumors, (C) compares the transplanted tumor volumes in each group, *p < 0.05, **p < 0.01, ***p < 0.001, ****p < 0.0001].

Following in vitro experiment, animal experiments were executed for PBS control group, low‐dose and high‐dose γδΤ cell groups. The therapeutic effect was assessed by comparing the tumor volumes across groups at day 21 (Figure 4). Both the low‐dose and high‐dose γδΤ cell groups demonstrated significant reductions in tumor volume compared to the PBS control group (*p* < 0.01), with the high‐dose group showing a more pronounced effect (*p* < 0.0001).

### 3.4 Impact of γδΤ Cell Immunotherapy on Body Weight

**FIGURE 5:**
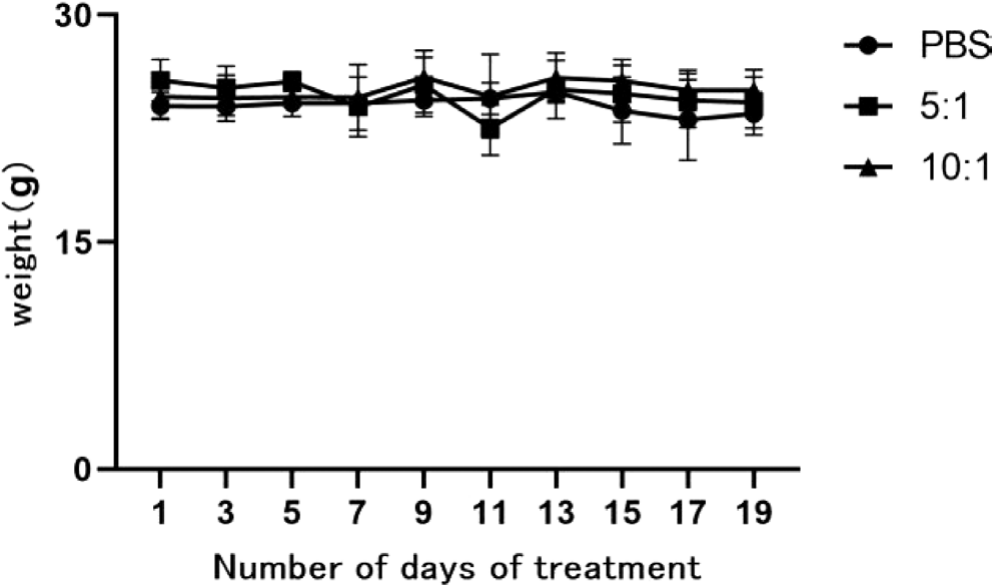
Body weight of mice in each group.

Following the initiation of adoptive γδΤ cell transfer, mice body weight was measured every 3‐day intervals (Figure 5). By the 21st day of treatment, mice in the PBS control group had an average weight of 23.5 ± 1.39 g, while those in the low‐dose and high‐dose γδΤ cell groups were 24.2 ± 1.70 g and 25.0 ± 1.3 g, respectively (*p* > 0.05).

### 3.5 Accumulation of γδΤ Cells in Mice Spleen

**FIGURE 6:**
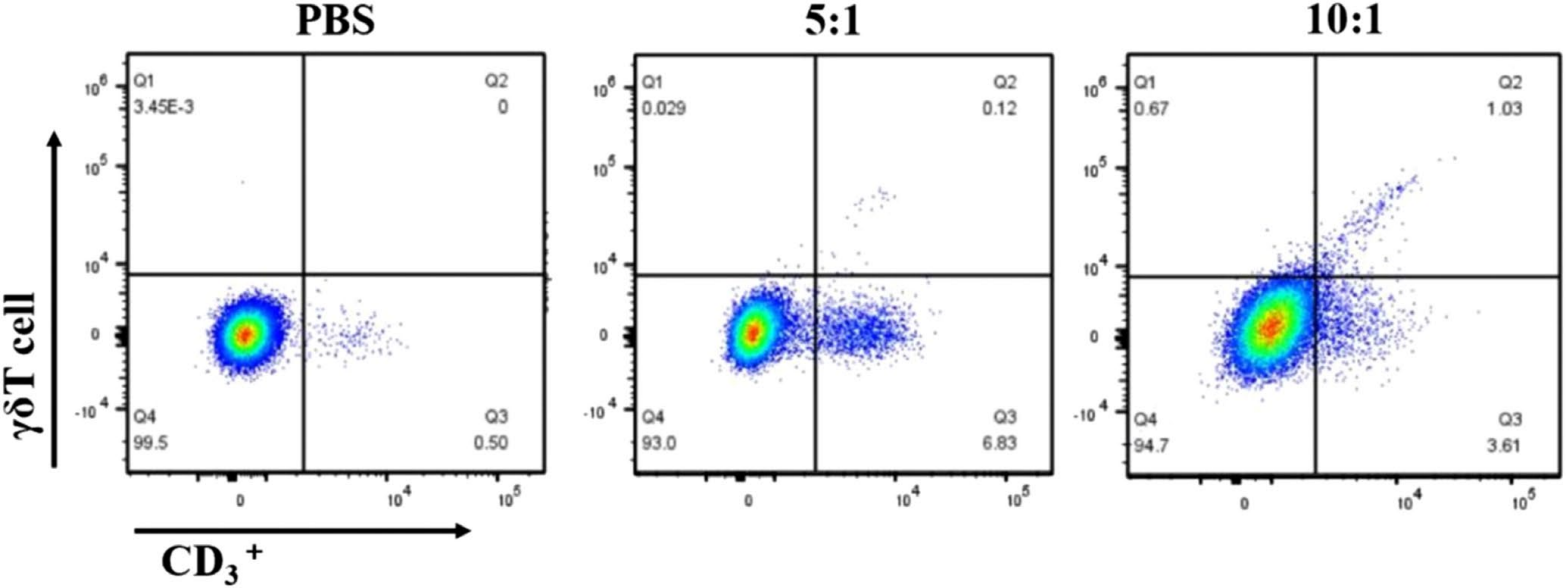
Accumulation of γδT cells in the spleen of mice.

Flow cytometry was used to measure the percentage of γδΤ cells in the mouse spleens following intervention (Figure 6). The proportions in the PBS control group, low‐dose γδΤ cell group, and high‐dose γδΤ cell group were 0.07 ± 0.03, 0.14 ± 0.02, and 1.02 ± 0.11, respectively. There were statistically significant differences among these groups (*p* < 0.01).

## 4 Discussion

In this study, focusing on γδΤ cells and their impact on TNBC, we established a mice model using MDA‐MB‐231 cells for γδΤ immunotherapy. Our in vitro findings revealed that γδΤ cells at an effective dose can eliminate TNBC cell lines, and in vivo, they are effective in tumor volume shrinkage.

γδT cells are a distinct subset of T lymphocytes that are important in antitumor immunity, functioning through direct cytotoxicity and indirect immunomodulation. Researchers on pancreatic ductal adenocarcinoma reveal that most of the tumor‐infiltrating T cells are γδT cells [28]. Significant changes in peripheral blood αβT and γδT lymphocytes in breast cancer patients before and after surgery were reported previously [29].

Studies using mouse models indicate that eliminating γδT cells or inhibiting PDL1 enhances infiltration and effectiveness of CD4+ and CD8+ T cells, slowing tumor growth [28]. These findings highlight the potent capabilities of γδT cells, underscoring their importance in cancer immunotherapy. In a study focusing on neoadjuvant therapy for breast cancer, a combination of letrozole and zoledronic acid was used to expand γδT cells in vivo, resulting in substantial benefits for patients [30]. In this study, zoledronic acid was used for in vivo activation of γδT cells, which was documented previously.

The use of γδT cells in adoptive immunotherapy, particularly after in vitro expansion, remains experimental. Aggarwal et al. isolated γδT cells from peripheral blood lymphocytes and cocultured them with various human breast cancer cell lines, demonstrating their significant inhibitory effect on the survival of breast cancer cells [31]. International studies suggest that allogeneic γδT cells from healthy donors have shown promising efficacy and safety in the treatment of advanced liver cancer or lung cancer through adoptive immunotherapy [32], but there is evidence supporting the effectiveness of γδT cell immunotherapy in TNBC that is still under investigation. In mouse xenograft tumor models, a significant reduction in tumor size, inducing apoptosis, and suppressed tumor angiogenesis was observed [33].

In this study, the variable cytotoxic effects of γδT cells on TNBC cells at different effector‐to‐target (E:T) ratios, particularly the enhanced cytotoxicity observed at a 10:1 ratio compared to 5:1 and 20:1 in a dose‐dependent way. Specifically, we hypothesize that the superior cytotoxicity at an E:T ratio of 10:1 may be attributed to an optimal balance between effector cell density and target cell accessibility. At a lower ratio (5:1), the reduced number of γδT cells might limit their ability to effectively engage and kill target cells. Conversely, at a higher ratio (20:1), excessive γδT cell density could lead to competition for target cells or even an inhibitory effect due to overcrowding that restricts cytotoxic activity. Additionally, γδT cells may exhibit dose‐dependent behavior where their activation and cytotoxic function are modulated by cell–cell interactions, cytokine release, or receptor dependency.

In this study, analysis reveals that the optimal E:T ratio for γδΤ cells to kill TNBC is 10:1, which guided us for animal model intervention. In subsequent experiments, we induced tumors in mice using MDA‐MB‐231 cells, followed by intravenous γδΤ cell therapy. After 21 days, both high and low doses of γδΤ cells significantly reduced tumor volume compared to the PBS control, with a notable accumulation of γδΤ cells in the spleen. It is speculated that γδΤ cell immunotherapy may emerge as a promising approach for TNBC treatment.

Using autologous T cells (i.e., the patient's own cells) would potentially enhance the effectiveness and feasibility of the treatment. However, our study focused on the killing ability of γδT cells against TNBC cell lines in vitro and verified the results in a mouse xenograft model using the MDA‐MB‐231 cell line. To further strengthen the clinical relevance, exploring the incorporated autologous γδT cell in experiments would be a valuable next step. This approach would not only help confirm the effectiveness of the treatment but also address potential issues related to immune rejection and graft‐versus‐host disease associated with allogenic T cells. Additional studies are necessary to elucidate how γδΤ cells can successfully infiltrate tumors and exert their antitumor effects within the intricate tumor microenvironment.

As documented, despite the limited numbers of γδT cells in peripheral blood, their role in tumor management as immunomodulators has gained increasing attention. In recent years, tumor immunotherapy has demonstrated its unique value and promise. In conclusion, the progress in γδT cell research provides new insight for developing innovative strategies for TNBC therapy. Further research on related mechanisms is necessary to optimize treatment approaches and improve the clinical efficacy of γδT cell immunotherapy for TNBC.

## Conflicts of Interest

The authors declare no conflicts of interest.

## Data Availability

The data that support the findings of this study are available from the corresponding author upon reasonable request.
